# Common Plant-Derived Terpenoids Present Increased Anti-Biofilm Potential against *Staphylococcus* Bacteria Compared to a Quaternary Ammonium Biocide

**DOI:** 10.3390/foods9060697

**Published:** 2020-06-01

**Authors:** Dimitra Kostoglou, Ioannis Protopappas, Efstathios Giaouris

**Affiliations:** Laboratory of Biology, Microbiology and Biotechnology of Foods, Department of Food Science and Nutrition, School of the Environment, University of the Aegean, GR-81 400 Myrina, Lemnos, Greece; dimitra_kostoglou@outlook.com.gr (D.K.); jproto2009@yahoo.gr (I.P.)

**Keywords:** *Staphylococcus aureus*, *S. epidermidis*, carvacrol, thymol, eugenol, benzalkonium chloride, biofilms, planktonic, disinfection, natural products

## Abstract

The antimicrobial actions of three common plant-derived terpenoids (i.e., carvacrol, thymol and eugenol) were compared to those of a typical quaternary ammonium biocide (i.e., benzalkonium chloride; BAC), against both planktonic and biofilm cells of two widespread *Staphylococcus* species (i.e., *S. aureus* and *S. epidermidis*). The minimum inhibitory and bactericidal concentrations (MICs, MBCs) of each compound against the planktonic cells of each species were initially determined, together with their minimum biofilm eradication concentrations (MBECs). Various concentrations of each compound were subsequently applied, for 6 min, against each type of cell, and survivors were enumerated by agar plating to calculate log reductions and determine the resistance coefficients (Rc) for each compound, as anti-biofilm effectiveness indicators. Sessile communities were always more resistant than planktonic ones, depending on the biocide and species. Although lower BAC concentrations were always needed to kill a specified population of either cell type compared to the terpenoids, for the latter, the required increases in their concentrations, to be equally effective against the biofilm cells with respect to the planktonic ones, were not as intense as those observed in the case of BAC, presenting thus significantly lower Rc. This indicates their significant anti-biofilm potential and advocate for their further promising use as anti-biofilm agents.

## 1. Introduction

*Staphylococcus aureus* is a common facultative anaerobic Gram-positive bacterial pathogen associated with a wide spectrum of minor to serious community and hospital-acquired infections. This non-motile, catalase and coagulase positive coccus is equipped with a tremendous range of virulence factors which allow its survival within the living host [1]. In addition, its ability to produce various heat stable enterotoxins in foodstuffs, makes staphylococcal foodborne intoxication one of the most common foodborne diseases worldwide [2]. Foods are usually contaminated through infected food handlers (via manual contact or their respiratory tract activity), while animal origin contamination is also frequent in products such as raw milk and cheeses [3]. *S. epidermidis* is usually a harmless commensal bacterium highly abundant on the human skin playing an important role in balancing the normal microflora. Nevertheless, this can still switch to an invasive lifestyle under certain predetermined conditions. Compared to *S. aureus*, this has, however, a more limited repertoire of virulence factors resulting in lower pathogenicity [1]. Nevertheless, this has still emerged as the most frequent cause of nosocomial infections primarily in patients with indwelling medical devices [4].

Both of these two species display a great ability to attach to various surfaces and create robust biofilms [5,6]. These surface-attached aggregated microbial communities are surrounded by a self-produced matrix of extracellular polymeric substances (EPS), allowing them to cope with many stresses and survive in inhospitable environments [7]. Indeed, biofilm formation is one of the most critical features that contributes to the success of these bacteria and is considered essential for the emergence of their pathogenesis and persistence [8]. Inside a biofilm, *Staphylococcus* bacteria (as well as other microbial human pathogens) can evade the host immune system and are in parallel protected against antibiotic treatment, making infections hard to eradicate [9]. In addition, pathogenic biofilms, formed on abiotic food-contact surfaces encountered within the food industry, including those being created by/containing staphylococci, allow embedded microorganisms to withstand killing action of common sanitizers, used at their recommended or even much higher concentrations, resulting in survival, cross contamination (through the ultimate dispersal of the remaining viable cells) and diseases transmission [10].

Therefore, there is currently an urgent demand to develop alternatives to conventional treatments (such as antibiotics and chemical sanitizers) to control unwanted biofilms in both healthcare and industrial environments [11]. In addition, due to the potential hazards of several synthetic biocides for both public health and the environment, novel eco-friendly approaches are nowadays preferred [12]. In this respect, numerous plant extracts and phytochemicals have been successfully evaluated as anti-biofilm agents in different model systems [13,14]. Besides their green status, these may present different modes of action from classical biocides, making them more efficient and probably helping to overcome the problem of resistance [15]. For instance, some phytocompounds have even been found to be capable of inhibiting biofilm formation in much lower concentrations than those required to inhibit planktonic growth, mainly through their interference with quorum sensing (QS) signaling pathways, something that seems to reduce the selective pressure exerted on the target microorganisms, in comparison with other antimicrobials, such as the antibiotics [16,17].

Carvacrol (CAR), thymol (THY) and eugenol (EUG) are natural terpenoids included in the most bioactive phytochemicals isolated from essential oils (EOs), all well-recognized for their wide spectrum of antimicrobial action, mainly due to their considerable deleterious actions on the cytoplasmic membranes [18]. Thus, CAR and THY are the main components occurring in EOs isolated from plants of the Lamiaceae family (e.g., oregano, thyme), which are commonly used as flavouring and preservative agents by the food industry processors, in commercial mosquito repellents, in aromatherapy, and in traditional medicine [19,20]. On the other hand, EUG is found in high concentrations in the EO of clove and has till now been applied in the agricultural, food, cosmetic and pharmaceutical industries [21]. All three of these plant metabolites are authorized as food flavourings across Europe [22], while EUG is also a permitted food additive by the U.S. Food and Drug Administration [23].

Benzalkonium chloride (BAC) is a synthetic quaternary ammonium compound (QAC) widely used as preservative, sanitizer and surface disinfectant in households, healthcare, agricultural and industrial settings, due to its broad antimicrobial spectrum against bacteria, fungi, and viruses [24]. In general, QACs, including BAC, exert their action by disrupting the bilayer and charge distribution of the cellular membranes, through the alkyl chains and charged nitrogen these are containing, respectively. Alarmingly, long-term low-dose microbial exposure to BAC might confer selective pressure and results in increased resistance both towards this compound, as well as other distinct chemicals, such as clinically relevant antibiotics, through cross-resistance mechanisms [25,26,27]. These last include changes in membrane composition, overexpression or modification of efflux pumps, downregulation of porins, horizontal transfer of stress response genes, biodegradation, and biofilm formation [24]. Not surprisingly, BAC-resistant staphylococci have been isolated from a variety of (seemingly distant) samples, such as environmental, hospital-acquired, animals, and foods, with several QAC resistance genes to have till now been identified, mainly and alarmingly easily transferable plasmid-borne ones encoding for efflux proteins [28,29,30,31]. Besides this great antimicrobial resistance problem, safety concerns regarding the use of BAC have also been emerged [32], with some countries to have already prohibited its use for some applications [24].

Considering all the above, it is evident that new antimicrobial agents that will be safe, cost-effective and in parallel exhibit as low as possible possibilities for resistance development are urgently required, especially to get rid of the most resistant biofilm-enclosed pathogenic microorganisms. For the effective development and application of such novel agents, it is, however, important to have previously compared their efficiency with the classically applied ones. Although several studies have been published in recent years related to the anti-biofilm action of many plant compounds, including CAR, THY and EUG, against various bacteria [33,34,35,36,37], including staphylococci [38,39,40,41], very few of them have compared their actions with those of standard chemical antimicrobials [42,43,44,45,46]. In addition, and to the best of our knowledge, no other study has been published comparing in parallel the efficiency of these three common plant-derived terpenoids (i.e., CAR, THY, and EUG) and of BAC against both planktonic and biofilm *Staphylococcus* bacteria or of other species.

Thus, the main objective of the present study was to compare the disinfection efficiencies of all these compounds (i.e., CAR, THY, EUG, and BAC) against both planktonic and biofilm cells of both *S. aureus* and *S. epidermidis*. For this, the minimum inhibitory and bactericidal concentrations (MICs, MBCs) of each compound against the planktonic cells of each bacterial species were initially determined, together with their minimum biofilm eradication concentrations (MBECs), by applying standard protocols for these purposes. Subsequently, both planktonic and biofilm cells of each species were exposed for 6 min to various concentrations (*n* = 3–4) of each compound, based on the previous determination of MBCs and MBECs, and the remaining viable cells were then enumerated by agar plating to calculate log reductions for each compound and at each tested concentration. This last made it possible to create the linear regression plots correlating these two parameters (log reductions vs. concentrations). These plots (for each compound, bacterial species, and cell type; *n* = 16) were finally used to accurately determine the resistance coefficients (Rc) of each compound against the biofilm cells of each species compared to its planktonic ones, as indicators for its anti-biofilm effectiveness. Results revealed the significant anti-biofilm potential of all three natural terpenoids (i.e., CAR, THY, and EUG) over the synthetic biocide (i.e., BAC), advocating for their further promising exploitation as anti-biofilm agents.

## 2. Materials and Methods

### 2.1. Chemicals and Stock Solutions

**Figure 1:**
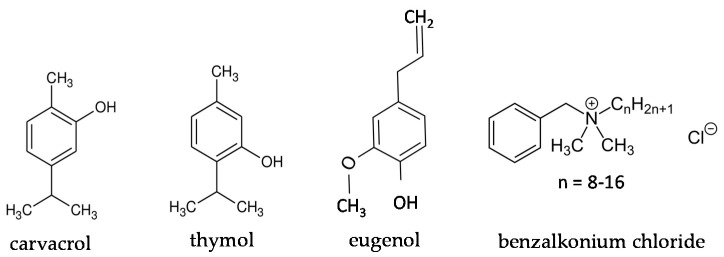
Chemical formulas of the four tested compounds.

Carvacrol (CAR), thymol (THY), eugenol (EUG) and benzalkonium chloride (BAC) were purchased from Sigma-Aldrich (liquid, ≥98%, molar mass: 150.22 g/mol, density: 0.976 g/mL; product code: W224502), Penta Chemicals (powder, >99.0%, molar mass: 150.22 g/mol; product code: 27450-30100), Alfa Aesar (liquid, ≥98.5%, molar mass: 164.21 g/mol, density: 1.068 g/mL; product code: A14332), and Acros Organics (liquid, alkyl distribution from C8H17 to C16H33, density: 0.98 g/mL; product code: 215411000), respectively. With respect to the terpenoids (i.e., CAR, THY, and EUG), two stock solutions for each one were prepared in absolute ethanol at 10% and 40% (*v*/*v* for CAR and EUG; *w*/*v* for THY), for subsequent use against planktonic and biofilm cells, respectively, following appropriate dilutions (see below), while the stock solution of BAC (1% *v*/*v*) was prepared in sterile distilled water. All stock solutions were maintained at −20 °C for up to 1 month. The chemical formulas of the four tested compounds are presented in Figure 1, while Table 1 summarizes their main physical and chemical properties, together with the correlations in the concentrations (for each compound) expressed in either as ppm or molarity (M), using the 0.1% (*v*/*v* or *w*/*v*) as a reference concentration.

### 2.2. Bacterial Strains and Preparation of the Working Saline Suspensions

The two bacterial strains used in this research were the *S. aureus* DFSN_B26, isolated in our lab from non-pasteurized milk cheese and the *S. epidermidis* DFSN_B4 (C5M6), originally isolated from fermenting grape juice and kindly provided by Professor G.-J. Nychas (Agricultural University of Athens, Greece). Before their use in the subsequent experiments, both strains were stored frozen (at −80 °C) in Tryptone Soy Broth (TSB; Lab M, Heywood, Lancashire, UK) containing 15% glycerol in cryovials and was then each one revivified by streaking a loopful of its frozen culture on to the surface of Tryptone Soy Agar (TSA; Lab M) and incubating at 37 °C for 24 h (precultures). Working cultures were prepared by inoculating, using a microbiological loop, cells of a district and well isolated colony from each preculture into 10 mL of fresh TSB and incubating at 37 °C for 18 h. Bacteria from each final working culture were collected by centrifugation (4000× *g* for 10 min at RT), washed twice with quarter-strength Ringer’s solution (Lab M), and finally suspended in the same solution, so as to display an absorbance at 600 nm (A_600 nm_) equal to 0.1 (*ca*. 10^7^ CFU/mL).

### 2.3. Determination of Minimum Inhibitory and Bactericidal Concentrations (MIC, MBC) of Each Compound against Planktonic Bacteria

The MIC of each compound (i.e., CAR, THY, EUG, and BAC) against the planktonic cells of each *Staphylococcus* species was determined using the broth microdilution method, as previously described [46]. Briefly, on the day of application, ten different concentrations for each compound were prepared by appropriately diluting its stock solution (i.e., 10% and 1%, for terpenoids and BAC, respectively) in fresh TSB. For terpenoids, the tested concentrations ranged from 19.5 to 10,000 ppm (two-fold dilutions), while for BAC those ranged from 1 to 10 ppm. Subsequently, 180 μL of each dilution were transferred to a well (in duplicate) of a sterile flat-bottomed 96-well polystyrene (PS) microtiter plate (transparent, hydrophobic, Ref 655101; Greiner bio-one GmbH, Frickenhausen, Germany) and 20 μL of a 10-fold dilution of the appropriate bacterial suspension (A_600 nm_ = 0.1) in quarter-strength Ringer’s solution were then added, so as to have an initial bacterial concentration in each well of ca. 10^5^ CFU/mL. Wells without bacteria and wells without any added compound served as negative and positive growth controls (for bacterial growth), respectively. The plates were sealed with parafilm and statically incubated at 37 °C for 24 h. The growth in each well was finally turbidimetrically assessed by naked eye observation and confirmed by measuring absorbances at 620 nm using a computer-controlled microplate reader (Halo Led 96; Dynamica Scientific Ltd., Livingston, UK). The MIC value was considered as the lowest concentration of each compound that totally inhibited the visible bacterial growth. To calculate MBCs, from all the wells showing no visible growth, 10 μL were aspirated and spotted on TSA and the number of colonies was counted following incubation at 37 °C for 48 h. MBC for each compound was defined as its lowest concentration, reducing the initial inoculum by at least three logs (i.e., no appearance of colonies).

### 2.4. Determination of Minimum Biofilm Eradication Concentration (MBEC) of Each Compound against Biofilm Bacteria

The MBEC of each compound (i.e., CAR, THY, EUG, and BAC) against the biofilm cells of each *Staphylococcus* species was determined following a previously described protocol, with some modifications [47]. Briefly, 200 μL of each bacterial suspension (A_600 nm_ = 0.1) were transferred into a well (in quadruplicate) of a sterile 96-well PS microtiter plate, and the plate was then statically incubated at 37 °C for 2 h, in order to allow bacteria to adhere to its surface. Following this adhesion step, the planktonic bacterial suspension was removed from each well, this was then washed with quarter-strength Ringer’s solution (to remove the loosely attached cells), and 200 μL of TSB containing 5% NaCl were added. The plate was then statically incubated at 37 °C for 48 h to allow biofilm growth. Following biofilm formation, the planktonic suspensions were removed, and each well was twice washed with quarter-strength Ringer’s solution (to remove the loosely attached cells). 200 μL of the appropriate antimicrobial solution were then added and left in contact for 6 min at 20 °C. Each compound was tested in five different concentrations, ranging from 8 to 128 × MBC (two-fold dilutions), which were all prepared in sterile distilled water starting from each stock solution (i.e., 40% and 1% for terpenoids and BAC, respectively). Sterile distilled water (also containing 6% *v*/*v* ethanol when CAR/THY were tested, or 24% *v*/*v* ethanol when EUG was tested) was used as the negative disinfection control. Those ethanol concentrations were included in the negative controls since were the maximum ones existing in the highest tested concentration for the terpenoids (i.e., 128 × MBC). Following disinfection, the antimicrobial solution was carefully removed from each well and this was then washed with quarter-strength Ringer’s solution, to remove any disinfectant residues. Subsequently, 200 μL of quarter-strength Ringer’s solution were added, and the strongly attached/biofilm bacteria were removed from the PS surface by thoroughly scratching with a plastic pipette tip, vortexed, serially diluted and finally enumerated by counting colonies on spot inoculated (10 μL) TSA plates following their incubation at 37 °C for 48 h. The MBEC for each compound was determined as its lowest concentration reducing biofilm cells by at least five logs (i.e., no appearance of colonies) with respect to the negative disinfection control.

### 2.5. Disinfection of Planktonic Bacteria

The disinfection of planktonic bacteria was carried out as previously described [46]. Briefly, 1 mL of each bacterial suspension (A_600 nm_ = 0.1) was centrifuged at 5000× *g* for 10 min at 20 °C, supernatant was discarded, and each pellet (ca. 10^7^ cells) was then suspended in 1 mL of the appropriate antimicrobial solution and left in contact for 6 min at 20 °C. Four different concentrations for each compound were tested (based on the previous MBC determination) and were all prepared in sterile distilled water by appropriately diluting its stock solution (i.e., 10% and 1% for terpenoids and BAC, respectively). Following disinfection, the antimicrobial action was interrupted by transferring a volume (1:9) to Dey-Engley neutralizing broth (Lab M) and leaving there for 10 min at 20 °C. Serial decimal dilutions were then prepared in quarter-strength Ringer’s solution, TSA plates were spot inoculated (10 μL) and colonies were counted following incubation at 37 °C for 48 h. Sterile distilled water (also containing 2.25% *v*/*v* ethanol when the terpenoids were tested) was used as the negative disinfection control. This ethanol concentration was included in the negative control since this was the maximum one with the highest preliminary tested concentrations for the terpenoids (i.e., 2500 ppm). For each compound and tested concentration, the logarithmic reduction (log_10_ CFU/mL) of cells following disinfection was calculated by subtracting the log_10_ of the survivors from that counted following disinfection with water (negative control).

### 2.6. Disinfection of Biofilm Bacteria

The disinfection of biofilm bacteria was carried out as previously described for the determination of the MBECs (Section 2.4), but this time, each terpenoid was tested in three different concentrations, while BAC was applied at four different concentrations (based on the previous MBEC determination). All these concentrations were lower than the MBECs, since the aim of this specific disinfection protocol was not to completely kill the cells, but to leave survivors for calculating log reductions at each tested concentration, so as to later be able to accurately calculate the resistance coefficients for each compound (Section 2.7). Sterile distilled water (also containing 0.4% *v*/*v* ethanol when the terpenoids were tested) was used as the negative disinfection control. This ethanol concentration was included in the negative control since was the maximum one existing in the highest tested concentration for the terpenoids (i.e., 2500 ppm). Survivors were again enumerated by counting colonies on spot inoculated (10 μL) TSA plates, while plate counts were converted to log_10_ CFU/cm^2^ before the calculation of log reductions (log_10_ CFU/cm^2^).

### 2.7. Calculation of Resistance Coefficients (Rc) of Each Compound against Biofilm Cells Compared to Planktonic Ones

**Figure 2:**
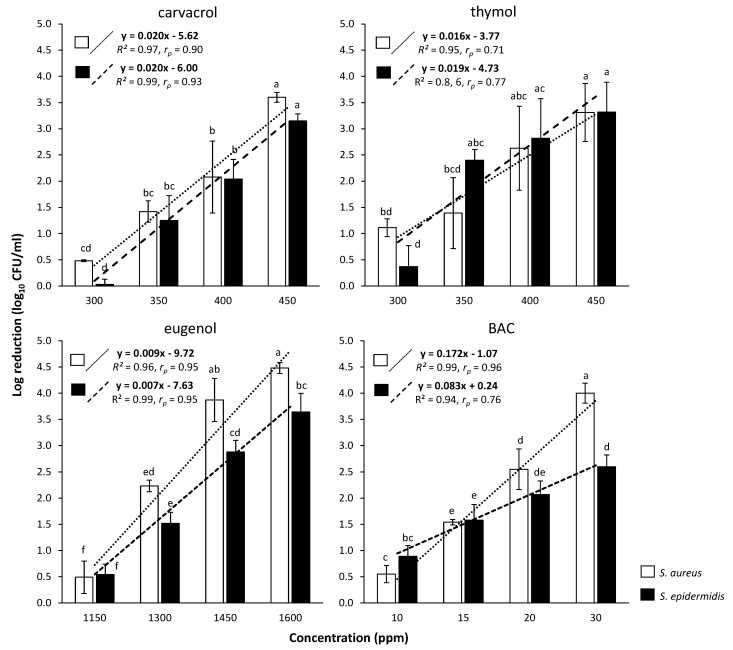
Log reductions (log_10_ CFU/mL) of planktonic cells for each bacterial species (□ *S. aureus*; ■ *S. epidermidis*) following 6 min exposure to each compound (i.e., CAR, THY, EUG, and BAC) applied at four different concentrations (ppm). The bars represent the mean values ± standard deviations. For each separate graph, mean values sharing at least one common letter shown above the bars are not significantly different (*p* > 0.05). Dotted lines illustrate linear regression correlations between the log reductions achieved (for each species) at each tested compound’s concentration. The mathematical equations of these regression plots, together with their regression coefficients (*R*^2^) and Pearson’s correlation coefficients (*r_p_*), are also shown.

**Figure 3:**
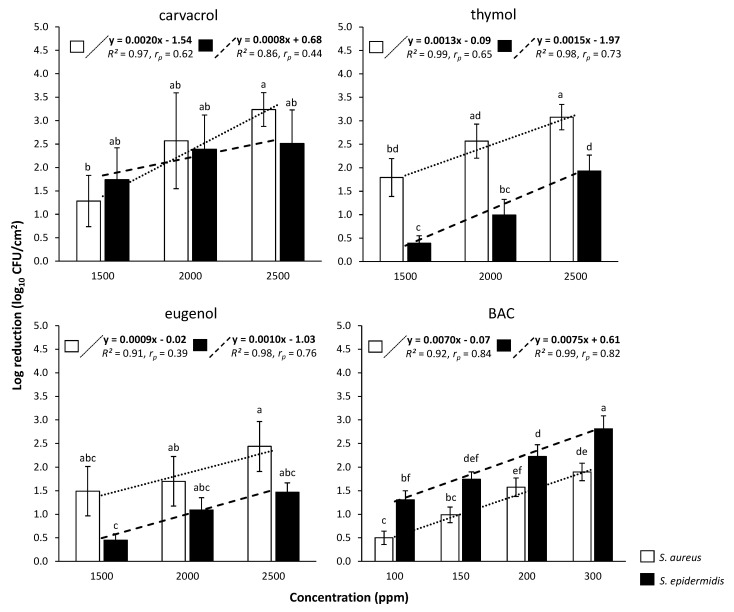
Log reductions (log_10_ CFU/cm^2^) of biofilm cells for each bacterial species (□ *S. aureus*; ■ *S. epidermidis*) following 6 min exposure to each compound (i.e., CAR, THY, EUG, and BAC) applied at different concentrations (ppm). The bars represent the mean values ± standard deviations. For each separate graph, mean values sharing at least one common letter shown above the bars are not significantly different (*p* > 0.05). Dotted lines illustrate linear regression correlations between the log reductions achieved (for each species) at each tested compound’s concentration. The mathematical equations of these regression plots, together with their regression coefficients (*R^2^*) and Pearson’s correlation coefficients (*r_p_*) are also shown.

To compare the antimicrobial action of each compound between the two cell types (i.e., planktonic, biofilm), its resistance coefficient was determined as the ratio of concentrations (Rc) required to achieve the same log reductions in both populations (C_biofilm_/C_planktonic_) [48]. Thus, for instance, a Rc equal to 10 means that a ten-fold more concentrated compound is needed to kill the same level of biofilm cells as planktonic. To accurately calculate Rc for each compound and against each bacterial species, a linear regression plot (standard curve) was constructed by plotting the log reductions achieved (for each cell type) at each tested compound’s concentration (based on the results of disinfection protocols presented in Section 2.5 and Section 2.6). The mathematical equations of each regression plot (*y* = a∙*x* + b; 16 equations in total i.e., 4 compounds × 2 bacterial species × 2 cell types; Figure 2 and Figure 3) were then used to calculate those concentrations required (*x*) to achieve prespecified log reductions (*y*). For this, at least 100 different log reduction values were considered for each linear regression equation (based on the total range of those covered by each standard curve). For each of those calculated log reduction—concentration combinations between the two cell types, the Rc value was obtained (by dividing the concentrations corresponding to the same log reduction: C_biofilm_/C_planktonic_) and finally the average Rc was determined for each compound and bacterial species. All calculations were done using the Excel^®^ module of the Microsoft^®^ Office 365 suite (Redmond, Washington, DC, USA).

### 2.8. Statistics

Each experiment was repeated at least three times using independent bacterial cultures. Plate counts were always transformed to logarithms before means and standard deviations were computed. All the disinfection data obtained for each compound (i.e., CAR, THY, EUG, and BAC), tested concentration (ppm), bacterial species (i.e., *S. aureus*, *S. epidermidis*), and cell type (i.e., planktonic, biofilm) were analysed by analysis of variance (ANOVA) to check for any significant effects of compound’s type, concentration and bacterial species on disinfection efficiency (expressed as log reduction), using the statistical software STATISTICA^®^ (StatSoft Inc.; Tulsa, OK 74104, USA). Following this analysis, least square means of log reductions were separated by Fisher’s least significant difference (LSD) test. The same test was also used to check for significant differences between the Rc values for each compound and bacterial species. Pearson correlation analysis was also applied to determine the significance of the correlations between log reductions (log_10_ CFU/mL or cm^2^) and tested concentrations (ppm) for each compound, bacterial species and cell type. All differences are reported at a significance level of 0.05.

## 3. Results

### 3.1. Determination of MICs, MBCs and MBECs of Each Compound

The MICs, MBCs and MBECs of each compound against each bacterial species are presented in Table 2. Thus, both CAR and THY presented an MIC against both species equal to 156.3 ppm, while eugenol was four times less efficient, presenting an MIC against both species equal to 625 ppm. As expected, BAC was capable of inhibiting bacterial growth at much lower concentrations, presenting an MIC against both species equal to just 3 ppm. At all cases, MBCs were two times more the respective MICs, confirming the bactericidal nature of all the compounds. With respect to the efficiency of the terpenoids (i.e., CAR, THY, and EUG) against the biofilm cells, someone observes that the MBECs against *S. aureus* were always two-fold lower compared to those observed against *S. epidermidis*, something that implies that *S. aureus* biofilm was less hard to eradicate using those compounds compared to that formed by *S. epidermidis*. On the contrary, the MBEC of BAC against *S. aureus* was two times more than that observed against *S. epidermidis*, indicating that *S. aureus* biofilm was less susceptible to BAC compared to *S. epidermidis* one. Similarly, to the antimicrobial efficiencies of each compound against the planktonic cells, BAC was again the most effective compound also against the biofilm cells, followed by CAR and THY (both these terpenoids present equal MBECs), while EUG was the least effective, needed for both species to be used in the highest concentration to eradicate their biofilm cells. However, it should be noted that the required increases in the compounds’ concentrations to be able to eradicate biofilm cells with regard the planktonic ones were always much lower for the terpenoids compared to BAC and for both bacterial species. This indicates that although terpenoids were always needed to be used at higher concentrations compared to BAC to kill the cells (either planktonic or biofilm), these still presented a better efficiency for destroying the biofilm cells than BAC when considering their “inherent” antimicrobial efficiencies against the planktonic bacteria. This last was more evident for EUG, than for the other two terpenoids (i.e., CAR, THY). Thus, EUG was capable of eradicating *S. aureus* biofilm population at just eight times more than its MBC (i.e., 10,000 ppm), whereas for the same to happen, BAC was needed to be used at 128 times more than its MBC (i.e., 768 ppm).

### 3.2. Comparative Evaluation of Disinfection Efficiencies of Each Compound against Planktonic and Biofilm Bacteria

The log reductions of planktonic (log_10_ CFU/mL) and biofilm (log_10_ CFU/cm^2^) cells of each species, following the 6 min exposure to each compound (i.e., CAR, THY, EUG, and BAC) being applied at different concentrations (ppm) are presented in Figure 2 and Figure 3, respectively. By observing these results, the following general remarks can be formulated. Firstly, log reductions always increased as the compounds’ concentrations increased. This means that more cells died when increasing a compound’s concentration; something that was rather expected (at least for the planktonic populations). However, it is worth noting that under the range of concentrations tested, the killing rates increased significantly faster for planktonic cells than for biofilm ones, highlighting the greater recalcitrance of the later. This is also clear when observing the concentrations needed for each compound to kill the same level of biofilm cells as planktonic. For instance, to kill 99% of planktonic *S. epidermidis* cells (i.e., to cause a 2-logs reduction), 20 ppm of BAC were enough (Figure 2), whereas this compound needed to be applied at 200 ppm (i.e., ten-fold more highly concentrated) to kill the same number of biofilm cells (Figure 3). Similarly, thymol at 450 ppm reduced planktonic *S. aureus* population by 99.9% (i.e., 3 logs), while this needed to be applied at 2500 ppm (i.e., more than five times more) to kill the same level of biofilm cells. Secondly, and in accordance to MBC and MBEC results previously presented (Table 2), EUG was the least effective compound, whereas BAC was the most effective one for both species and cell types (i.e., planktonic, biofilm). Thus, for instance, 1450 ppm of EUG were needed to reduce planktonic *S. aureus* population by 4 logs, whereas 30 ppm of BAC were enough for the same effect (Figure 2). Similarly, biofilm population of the same species was reduced by 1.5 log upon applying 200 ppm of BAC, whereas for the same log reduction EUG needed to be applied at ten times higher concentration (2000 ppm) (Figure 3). Thirdly, the resistance of biofilm cells seems to be significantly influenced by the forming species and compound tested. Thus, *S. epidermidis* biofilm was always more resistant (i.e., presenting lower log reductions) to both THY and EUG compared to the *S. aureus* one. However, the opposite occurred when these biofilms were exposed to BAC, with *S. aureus* always presenting lower log reductions than *S. epidermidis*. This last observation is in full accordance with the MBEC results previously presented (Table 2).

**Figure 4:**
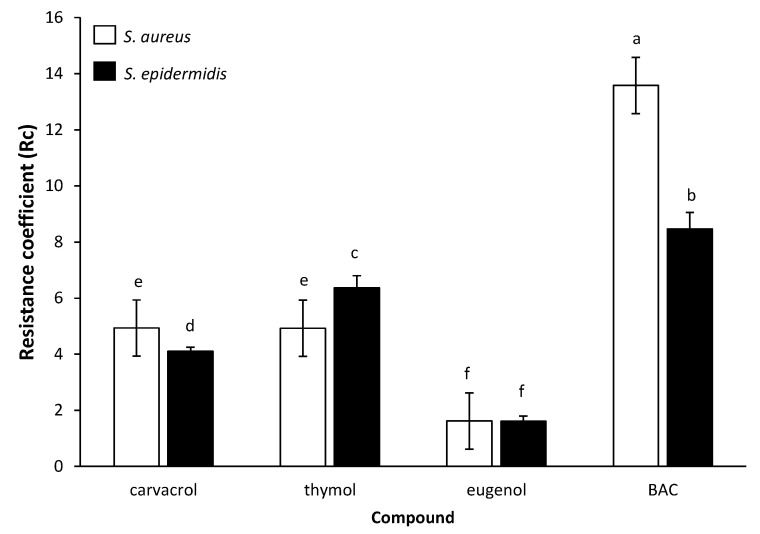
Resistance coefficients (Rc) of each compound for each bacterial species (□ *S. aureus*; ■ *S. epidermidis*). The bars represent the mean values ± standard deviations. Mean values sharing at least one common letter shown above the bars are not significantly different (*p* > 0.05).

To accurately compare and easily perceive the efficiency of each compound against each cell type (i.e., planktonic vs. biofilm), its resistance coefficient (Rc) was determined, based on the results of log reductions for each cell type following disinfection and the respective regression plots (Figure 2 and Figure 3). The calculated Rc values are presented in Figure 4. Thus, the quaternary ammonium compound BAC was found to exhibit the highest Rc values equal to 13.6 and 8.5 against *S. aureus* and *S. epidermidis*, respectively. This means that this compound needed to be applied at concentrations 13.6 and 8.5 times higher to kill the same numbers of biofilm cells as the planktonic ones. On the contrary, EUG exhibited the lowest Rc values (i.e., 1.6 against both bacterial species), highlight its almost similar efficiency against both cell types. The other two terpenoids (i.e., CAR, THY) presented Rc values near to 4 (with some minor differences between them and depending on the bacterial species), meaning that these needed to be applied in concentrations approximately four times greater against biofilm cells to achieve similar log reductions with respect to planktonic ones. This remarkable potential of all three terpenoids against the biofilm cells was also previously noticed upon presenting the MBEC results (Table 2).

## 4. Discussion

To comparatively evaluate the disinfection efficiencies of each compound (i.e., CAR, THY, EUG, and BAC) against each cell type (i.e., planktonic, biofilm) and for each bacterial species (i.e., *S. aureus*, *S. epidermidis*), their MICs, MBCs and MBECs were initially determined following some standard protocols (Table 2). It was revealed that for both cell types and species, the synthetic biocide BAC was quite a bit more efficient than the three plant-derived terpenoids, presenting the lowest MICs, MBCs and MBECs. The identical MIC value for both staphylococci (i.e., 3 ppm) reveals their intermediate planktonic resistance, according to the Clinical and Laboratory Standards Institute guidelines [49], which define staphylococci as being resistant to BAC upon presenting an MIC greater than 3 ppm. On the contrary, the least effective compound was EUG, presenting the highest MICs, MBCs and MBECs. Compared to those, CAR and THY displayed intermediate and equal efficiencies. These results were rather expected based on the rich available literature concerning the antimicrobial actions of these compounds. Thus, like our results, the MIC of EUG was found to be four times greater than that of CAR against an *S. aureus* strain (1000 and 250 ppm, respectively), previously determined with a broth liquid method where sterile filter papers impregnated with each compound had been placed into inoculated broth tube cultures [50]. The slight differences between those MIC values and ours could just be due to the different strain and method followed to determine these values.

More generally, the lower efficiency of EUG compared to either CAR or THY should be attributed to its lower hydrophobicity with respect to the latter compounds, considering that the most hydrophobic cyclic hydrocarbons are generally reported to present more toxic effects and as such be more antimicrobial [51]. In addition, CAR and THY are isomeric compounds that only differ in the position of their free hydroxyl group, and they can both release the proton of this group more easily than EUG, which also presents a methoxyl group in ortho position (Figure 1). This better proton exchange activity is believed to allow CAR and THY to more easily collapse the proton gradient (motive force) across the cytoplasmic membrane [50]. Relatively close to the present results, the MIC of EUG against *S. aureus* strains recovered from the milk of cows with subclinical mastitis was 392 ppm [52].

In a previous similar study evaluating the susceptibility of 26 methicillin-susceptible (MSS) and 21 methicillin-resistant staphylococci (MRS) to CAR and THY using an agar dilution method, MIC values of 150–300 ppm and 300–600 ppm were reported for CAR and THY, respectively, with no significant differences between MSS and MRS regarding their susceptibility [53]. Another study also found that the MICs of THY against 6 *S. aureus* strains (ATCC29213 and 5 MRSA strains) ranged from 250 to 375 ppm, with the MBECs also found to be two- to three-fold higher than those (530–1070 ppm) [54]. In another previous study evaluating the effect of CAR and THY on biofilm-grown *S. aureus* and *S. epidermidis* strains (6 strains per each species), as well as their effects on biofilm formation, it was found that for most of the strains tested, the biofilm eradication concentrations (i.e., 1250–5000 ppm) were two- to four-fold greater than the concentrations required to inhibit planktonic growth [55]. However, it should be noted that in all those previous studies, the protocol used to form biofilms (i.e., in TSB containing 0.25–1% *v*/*v* glucose at 37 °C for 24 h, with no initial attachment step) was quite different from the one here applied, while in addition and more importantly the terpenoids had been left to act for 24 h, whereas a short 6-min exposure was applied here, thus making any attempted comparison risky. Thus, in the present study, biofilms of both species were left to be formed in a general purpose medium (i.e., TSB) and in the presence of high salt concentration (i.e., 5% *v*/*v* NaCl), at 37 °C for 48 h (following a 2-h initial attachment step in saline), since it is known that high osmolarity usually induces the biofilm-forming potential of staphylococci, mainly through the increase in the expression of several biofilm-associated genes this can provoke [56,57]. Preliminary experiments by our group have also confirmed this positive influence of NaCl on biofilm formation by the two staphylococci strains applied here (results not shown). In addition, the short exposure time (i.e., 6 min) was selected here to imitate conditions that could be applied within the food industry or for surface disinfection in other environments, such as the clinical ones, where a short disinfection period is usually desired. In this direction, standard protocols approved for the evaluation of the bactericidal activity of chemical disinfectants also propose exposure times ranging from 1 to 60 min (e.g., EN 1276) [58].

All three terpenoids tested here (i.e., CAR, THY, and EUG), being phenolic compounds with both hydrophilic and hydrophobic properties, are known to be capable of interacting with the lipid bilayer of the cytoplasmic membranes, provoking the loss of their integrity, disruption of the proton’s motive force, impairment of intracellular pH homeostasis, and leakage of cellular material including ATP [50]. In addition, their relative hydrophilic nature conferred by the free hydroxyl group these are all containing, is believed to further allow their ease diffusion through the polar polysaccharide biofilm matrix, and as such the efficient killing of the enclosed bacteria [50]. Interestingly, time-lapse confocal laser scanning microscopy (CLSM) has previously revealed the significant advantage of another plant mixture rich in CAR and also containing both THY and EUG (i.e., the hydrosol of the Mediterranean spice *Thymbra capitata*) for easily penetrating into the three-dimensional (3D) biofilm structure of *Salmonella* Typhimurium and quickly killing the cells, when compared to BAC [42]. In that study, the Rc value for that hydrosol mixture was found to be quite low (1.6), a value equal to that found in our study for EUG. On the other hand, in that previous study BAC was found to present an Rc value equal to 208.3, whereas an average Rc value of 11.1 (i.e., 13.6 and 8.5 for *S. aureus* and *S. epidermidis*, respectively) was determined here for this compound (Figure 4). In the literature, the Rc values for the BAC range significantly from 10 to 1000, but in most cases, these surpass 50 [48]. It is surely difficult, if not impossible, for someone to compare results obtained in different studies, due to the large variations in the experimental setup (e.g., different bacterial strains, support materials, growth media, biofilm forming procedure, incubation temperatures and times), which can drastically influence the phenotypic behaviour (including resistance) of the formed biofilms. Disinfection exposure times also vary greatly between the different studies.

The lower Rc values of EUG found here against both bacterial species compared to either CAR or THY, and as thus its relative better anti-biofilm efficiency when also considering the “inherent” antimicrobial action of all these terpenoids against planktonic cells, may be attributed to its lower hydrophobicity, and as thus its better solubility and diffusion in the water containing EPS biofilm matrixes [50]. This is surely something that deserves to be further investigated and verified through microscopy. In a planktonic system, however, where EPS are either absent or encountered in low amounts, the higher hydrophobicity of both CAR and THY, together with their better proton release abilities, seems to increase their toxic effects against the freely accessible bacteria, as previously reported [50]. It is also worth noting that the Rc values determined here for both CAR and THY (i.e., from 4.1 to 6.4, depending on the compound and bacterial species; Figure 4), are close enough to those previously reported in the literature for these two compounds [48]. It should still be noted that the approach we here followed to calculate Rc took into account a large range of different log reduction-concentration combinations (>100), through the previous construction of the regression plots significantly correlating these two interrelated parameters (Figure 2 and Figure 3), whereas in all the previous studies, the Rc values were usually calculated based on either a limited number of tested concentrations or solely through the comparison of MBC and MBEC results. Our more sophisticated approach not only confirmed the MBC and MBEC results determined here (Table 2), but also seems to more accurately calculate the Rc values for each compound (as reliable anti-biofilm effectiveness indicators).

In another study comparing the antimicrobial action of CAR to that of a peroxide-based commercial sanitizer at various stages of dual-species biofilm development by *S. aureus* and *S*. *Typhimurium* (in a constant-depth film fermenter system for up to 21 days), it was found that the commercial sanitizer was more biocidal than CAR only during early biofilm development (<3 days), whereas the natural terpenoid outmatched it when the biofilm had reached a quasi-steady state [44]. This last point undoubtedly further highlights the importance of biofilm maturation stage when someone evaluates the effectiveness of antimicrobial treatments. In our study, biofilms were left to be formed for 48 h under static conditions, resulting in both species achieving biofilm populations of over 10^7^ CFU/cm^2^ by the end of incubation (just before disinfection; results not shown). Such cell-concentration levels are considered adequate for sufficient (mature) biofilm formation (and not just individual cells attachment), with many other previous studies having left staphylococci to form biofilms on PS microtiter plates for just 24 h before further experimentation [59,60,61]. However, we still do not have any other further info regarding the structure and composition of the extracellular material of the biofilms formed here or whether and in which way these characteristics, together with the variation in biofilm incubation time (or many other parameters that could potentially influence biofilm growth), could affect the resistance of the enclosed bacteria to the tested antimicrobials. Nevertheless, the higher resistance of *S. epidermidis* biofilms to all three terpenoids tested here compared to those formed by *S. aureus*, together with the increased resistance of the latter to BAC (Table 2 and Figure 3), should probably imply a different matrix structure and/or composition of the biofilms formed by these two distinct species, given their similar planktonic resistance (Table 2 and Figure 2). The important roles of biofilm matrix on the overall physiology of the enclosed microorganisms and their interactions with the environment (including disinfectants) have also been well documented in the literature [62]. Not only does its synthesis depend on the involved microbial species, but in addition, its exact composition and conformation can considerably vary even within the same species, depending on the strain and the prevailing environmental conditions [63].

Obviously, the high heterogeneity that biofilms may present, even those formed by the same microorganism under different environmental conditions, is something that should be always considered when studying biofilms and their resistance, since it could drastically influence the results obtained. Future studies also employing different strains of various species, being left to develop mixed-culture sessile communities, could also further increase our knowledge of the efficiency of novel anti-biofilms approaches, and their superiority (if any) over the traditional ones. We should not forget that in nature and in several other habitats as well (e.g., food industry, healthcare), biofilms may be composed of a variety of different microorganisms interacting in quite complex ways with each other [64]. All these interactions could ultimately leave their notorious imprint on biofilm robustness and resistance.

## 5. Conclusions

The three plant-derived terpenoids (i.e., CAR, THY, and EUG) were found to present increased anti-biofilm potential against staphylococci, when compared to BAC. Thus, the required increases in their concentrations to be equally effective against biofilm cells as they are against the planktonic ones were always much lower compared to the synthetic biocide. This was more evident for EUG, which was found to present a very low Rc (i.e., 1.6), revealing almost similar effectiveness against both cell types, quite probably due to its good diffusion through the biofilm matrix. These results confirm and increase our knowledge of the significant bactericidal and in parallel anti-biofilm actions of all these three terpenoids, advocating for their further use as promising alternatives or supplementary agents (e.g., application together with antibiotics or other sanitizers) for dealing with biofilm-enclosed resistant microorganisms and as thus improve the quality of modern human life.

## Figures and Tables

**Table 1:** Main physical and chemical properties of the four tested compounds, together with the correlations in concentrations (for each compound) expressed in either as ppm or molarity (M), using the 0.1% (*v*/*v* or *w*/*v*) as a reference concentration.

Compound	Physical Form (20 °C)	Molar Mass (g/mol)	Density (g/mL)	Concentration		
				%	ppm	M (mol/L)
carvacrol	liquid	150.22	0.976	0.1 (*v*/*v*)	1000	0.00650
thymol	powder	150.22	unknown ^1^	0.1 (*w*/*v*)	1000	0.00666
eugenol	liquid	164.21	1.068	0.1 (*v*/*v*)	1000	0.00650
BAC	liquid	unknown ^2^	0.98	0.1 (*v*/*v*)	1000	unknown ^2^

^1^ Not provided by the manufacturer; ^2^ BAC was provided a mixture of QACs with different lengths for the alkyl chain (ranging from C8 to C16).

**Table 2:** MICs, MBCs and MBECs of each compound against each bacterial species.

Compound	MIC ^1^		MBC ^1^		MBEC ^1^	
	*S. aureus*	*S. epidermidis*	*S. aureus*	*S. epidermidis*	*S. aureus*	*S. epidermidis*
carvacrol	156.3	156.3	312.5 (2 × MIC)	312.5 (2 × MIC)	5000 (16 × MBC)	10,000 (32 × MBC)
thymol	156.3	156.3	312.5 (2 × MIC)	312.5 (2 × MIC)	5000 (16 × MBC)	10,000 (32 × MBC)
eugenol	625	625	1250 (2 × MIC)	1250 (2 × MIC)	10,000 (8 × MBC)	20,000 (16 × MBC)
BAC	3	3	6 (2 × MIC)	6 (2 × MIC)	768 (128 × MBC)	384 (64 × MBC)

^1^ All concentrations are expressed as ppm (1000 ppm = 0.1% *v*/*v*).
